# A Happy Result

**Published:** 1889-01-19

**Authors:** 


### A HAPPY RESULT.

ON November 8th, at a meeting of the Northumberland and Durham Medical Society, a little girl aged nine was shown to the members, on whom a most interesting operation had been successfully performed. When two years old she had had measles. Following the attack there was, as there often is, a profuse and prolonged discharge of matter from the right ear. Gradually the mouth began to show signs of difficulty in opening, and in six months it entirely closed. It remained immovably closed for six years. The hospital surgeon, when he saw the case, did not attempt, as a "bonesetter" might have done, to pull the mouth violently open. If he had he would most probably have killed the child, as "bonesetters" have often done. He behaved rationally. He cut down upon the head of the jawbone, and looked and felt for the real cause. It was soon found. The head of the bone had become diseased at the time of the "measles," and had gradually disappeared, first by suppuration and then by absorption. The inflammation then set up had caused the jawbone to grow to the socket, and so had of course immovably fixed the whole jaw and firmly closed the mouth. The fixed portion of the bone was at once removed without danger, and the girl rapidly got well. She can now open her mouth freely, and, what may be almost as important to her, being of the tender sex, can also use her tongue with equal freedom.

